# Post-Mortem Findings in Measles-Bronchopneumonia and Other Acute Infections

**Published:** 1918-11

**Authors:** 


***Post-Mortem Findings in Measles-Bronchopneumonia and other Acute Infections.*** By BALDWIN LUCKE. *The Journal of the American Medical Association,* June 29, 1918.

The author gives his experience with 62 post-mortem examinations performed during the past winter at Camp Zachary Taylor, Ky.

Bronchopneumonia caused 35 deaths out of the 62 cases, and lesions of the lungs and meninges contributed 51 primary lethal cases.

*Bronchopneumonia*, was preceded by measles in 16 cases out of 35. The streptococcus was isolated in 13 cases; not isolated in 22. In the latter group the pneumococcus was frequently found.

*Types of Bronchopneumonia.* Four anatomic types were found with such regularity that they were classified as Types Ia, Ib, II and III. The two first were mainly found in bronchopneumonia following measles; the two last in hemolytic streptococcic infection.

The principal features of Types Ia and Ib are marked edema, the discrete character of the consolidation, *their distinct peribronchial situation*, and their occurrence in all lobes. The totality of the consolidated areas would equal from 1/3 to 2/3 of the entire pulmonary substance. A purulent bronchitis accompanied the lesions.

Type II resembles the ordinary type, except for the great frequency with which empyema occurs, and the hemorrhagic character of the bronchitis.

Type III resembles pseudolobar lobular pneumonia. A large part of a lobe is consolidated, granularity being less distinct than that of lobar pneumonia.

*Measles-Bronchopneumonia.* One of the dominant symptoms of measles is bronchitis: appearing early and often persisting. The author is inclined to think that true measles-bronchopneumonia exists, and is due to the virus of measles. It is that kind of bronchopneumonia that has been described under Types Ia and Ib. Cases of these types were detected mostly in the early post-mortem examinations, before the epidemic of streptococcus broke out in the camp. Since it is a fact that pneumococci and streptococci coexist constantly in the throat, secondary invasion may take place, and both diseases develop in some cases. If these organisms possess marked virulence and invasive powers, and if they intrude early in the primary disease, the anatomic picture will be determined by this invading bacteria, and the possible influence of the measles virus may be obscured. The diagnosis will be of a *true* streptococcic or pneumococcic bronchopneumonia. If, on the other hand, the invading organisms enter the system late in the disease, are few in number, and do not possess marked virulence, they will influence very little, if at all, the pathologic process, and a true *measles bronchopneumonia* will develop.

*Streptococcic Bronchopneumonia.* The majority of these cases were of Type II. The bronchi showed hemorrhagic inflammation; empyema was most frequent. The spleen presented the picture of an acute splenitis, often with hemorrhages. Otitis media, mastoiditis, and other complications occurred frequently. Sub-serous, pin-point hemorrhages could be found on all visceral surfaces.

*Coexistent Lobar and Bronchopneumonia.* In three cases both infections coexisted. One case presented a peculiar picture. The entire right lung was consolidated; from the upper and middle lobes pneumococcus cultures were made; from the lower lobe hemolytic streptococci were isolated. The streptococcic infection was most likely superadded to the pneumococcic involvement.

*Empyema* was present in 21 out of 52 cases. Clinically a " cold " or " sore throat " frequently preceded the empyema by a few days. The following symptoms were noted : a severe chill lasting sometimes over an hour, followed by cough, pain in the side, fever, dyspnea. The empyema was of unusually rapid occurrence, often developing over night. Organisms found were : hemolytic streptococci, fourteen; pneumococci, five. In four cases both pleural cavities were affected. Interlobar empyema was only found once.

The quantities of fluid varied between 100 and 2,500 cc.; it was distinctly turbid, in most cases frankly purulent. In all cases the pleural surfaces were coated with a yellow, soft exudate, from 3 to 10 mm. thick, binding the lung lightly to the chest wall here and there. If the exudate process had existed for some time, the exudate was much tougher, adhesions firmer, and empyema pockets were found. This seems to prove that as often as possible early operations should be practised in order to offer the best chances of complete drainage.

In several instances the infected lung was no larger than a fist. The uninvolved side showed compensatory inflation.

*Acute Pericarditis* occurred in 10 cases. Lobar pneumonia coexisted in one of these, bronchopneumonia in six, and in three instances the lung did not show any inflammatory process. In all but one case the pericardial inflammation was associated with empyema.

*Changes in the Cardiovascular and the Abdominal Viscera.* The heart generally showed right-sided dilatation and cloudy swelling. The aorta presented atheroma in a very high percentage of cases. The liver usually showed cloudy swelling. The gastro-intestinal tract presented no noteworthy changes.

*Meningitis.* Meningococcic, pneumococcic, and streptococcic meningitis were all observed, and also one case of marked hemorrhagic encephalitis as a complication to bronchopneumonia. In this latter case the brain showed extensive subpial hemorrhagic extravasations. The large veins were greatly congested and in the cut surfaces unusual numbers of bleeding points were present.

